# Unveiling new perspectives about the onset of neurological and cognitive deficits in cerebral malaria: exploring cellular and neurochemical mechanisms

**DOI:** 10.3389/fcimb.2025.1506282

**Published:** 2025-02-06

**Authors:** Renato M. S. de Lima, Luana K. R. Leão, Luana C. Martins, Adelaide da C. Fonseca Passos, Evander de J. Oliveira Batista, Anderson M. Herculano, Karen R. H. M. Oliveira

**Affiliations:** ^1^ Laboratory of Experimental Neuropharmacology, Biological Science Institute, Federal University of Pará, Belém, Brazil; ^2^ Laboratory of Protozoology, Tropical Medicine Nucleus, Federal University of Pará, Belém, Brazil

**Keywords:** cerebral malaria, neurotransmitters, neurological sequelae, cytoadherence, neuroinflammation

## Abstract

Cerebral malaria is the most severe and lethal complication caused by *Plasmodium falciparum* infection, leading to critical neurological impairments and long-term cognitive, behavioral, and neurological sequelae in survivors, particularly affecting children under the age of five. Various hypotheses have been proposed to explain the neurological syndrome associated to cerebral malaria condition, including vascular occlusion and sequestration, cytokine storm or inflammatory response, or a combination of these mechanisms and despite extensive research and a growing range of scientific information, the precise pathophysiological mechanism remains poorly understood. In this sense, this review aims to explore the neurological impairment in cerebral malaria and elucidate novel mechanisms to explain the severity of this disease. Recent evidence implicates glutamate and glutamatergic pathways in the onset of cerebral malaria, alongside the impairments in the metabolic activity of other molecules such as dopamine and kynurenic acid. These neurotransmitters pathways may play a crucial role in the pathogenesis of cerebral malaria, potentially interacting with other molecular players. By enhancing our understanding in the pathophysiology of cerebral malaria, this article seeks to explore new hypotheses regarding the involvement of neurotransmitters and their interactions with other molecular targets, thereby contributing to the overall pathology of cerebral malaria.

## 1 Introduction

Malaria remains as a critical infectious disease with severe public health implications, caused by protozoa of the genus *Plasmodium* and transmitted through the bites of female *Anopheles* mosquitoes during their blood-feeding process (Khanam, 2017; WHO, 2022). Among more than 100 species of *Plasmodium*, seven can infect humans, although *P. falciparum* and *P. vivax* are the most common. Annually, more than 247 million new cases of malaria are reported worldwide, and even though it is a preventable and treatable disease, about 619,000 people die yearly (WHO, 2022). Although the African continent accounts for 95% of cases, malaria has a wide distribution in 84 endemic countries located in Asia, India, Central America and South America (WHO, 2022). Two-thirds of the number of deaths are children under five years old, with 94% of cases being on the African continent with a higher incidence of malaria induced by *P. falciparum* (WHO, 2020) that can trigger the most severe aspect of the disease, making it potentially fatal (Miller et al., 2002; Tuteja, 2007; McQueen and McKenzie, 2008; Kantele and Jokiranta, 2011; White et al., 2014). Infections caused by *P. falciparum* are linked to a severe complication impacting the central nervous system known as cerebral malaria (CM). Especially in tropical regions, cerebral malaria diagnosis is particularly associated with several neurological signs. CM is diagnosed in around 1% of *P. falciparum* cases and its mortality rate fluctuates between 15% and 25%. Survivors often face acute or persistent physical disabilities and neurological syndromes, persisting even after the infection has been treated (Daniyan et al., 2022; WHO, 2022).

Obstacles faced by countries to eliminate malaria are related to the parasite resistance to treatment and mosquito resistance to insecticides (WHO, 2017; Daniyan, 2021; Sabina, 2017). Furthermore, severe complications of the disease exacerbate the malaria issue, primarily due to limited understanding of their pathogenesis. Consequently, numerous studies are being conducted to elucidate these complications. Regarding the establishment of neurological complications associated with cerebral malaria, a comprehensive understanding of all underlying mechanisms is crucial as the disease complications endure even after the treatment of the *Plasmodium* infection (Desruisseaux et al., 2010; Ghazanfari et al., 2018). Therefore, this review aims to elucidate the latest hypotheses concerning the pivotal role of cellular mechanism and cerebral neurochemistry in the pathogenesis of cerebral malaria.

## 2 Clinical insights into cerebral malaria

Understanding the clinical aspects of the disease involves critical stages of the complex *Plasmodium* life cycle, implicating both intracellular and extracellular phases, which results in the disease rapid progression. Human malaria infection begins when sporozoites are inoculated by mosquitoes, initiating the asexual phase of the cycle, which includes the pre-erythrocytic and erythrocytic stages. In the pre-erythrocytic phase, sporozoites migrate to the liver and infect hepatocytes, where they proliferate to produce approximately 30,000 merozoites. These merozoites then infect erythrocytes, marking the onset of the asexual erythrocytic phase (Tuteja, 2007). During the asexual erythrocytic phase, merozoites progress through three stages: ring, trophozoite, and schizont. The ring stage represents the young trophozoite with a ring-shaped structure. The trophozoite matures with increased metabolic activity, including hemoglobin ingestion and proteolysis, leading to schizont formation characterized by multiple cell cycles without cell division (Natarajan et al., 2001; Miller et al., 2002; Tuteja, 2007).

The first clinical symptoms of malaria are nonspecific, initiated from the rupture of parasitized erythrocytes, which release antigens into the bloodstream, activating the host immune response (De Souza and Riley, 2002). Among them, febrile episodes, chills, tremors, headache, nausea, vomiting and other clinical signs stand out, however, in the case of *P. falciparum* infection, severe conditions such as anemia, respiratory distress, organ dysfunction and malaria brain damage are also common, which potentially can lead to cognitive deficits, declining the survivors overall life quality (Hunt and Grau, 2003; Combes et al., 2006; Carvalho et al., 2014; Martins et al., 2009a; Trampuz et al., 2003; Medana and Turner, 2006). Given the impacts of cerebral malaria on patients, multiple approaches are being explored to unravel all the counterpoints involving the pathogenesis of these neurological complications.

Human cerebral malaria (HCM) is a neurological disorder resulting from complications of malaria caused by *Plasmodium falciparum* infection, which commonly affects adolescents and children (Desruisseaux et al., 2010). According to the World Malaria Report (WHO, 2022), 610,000 patients died from malaria, with CM accounting for 90% of deaths. CM complications include high fever, recurrent chills, lethargy, severe headache, delirium, seizures, manifests as encephalopathy with a coma state, accompanied by neurological disorders stemming from hemorrhagic events (Bangirana et al., 2014; Lou et al., 2001; Rosa-Gonçalves et al., 2022).

Despite infection treatment, approximately a quarter of surviving patients experience enduring neurologic and cognitive deficits, including behavioral abnormalities, epilepsy, learning and memory deficits, as well as impaired motor functions, both short and long after clinical episodes (MacCormick et al., 2014; Idro et al., 2006; Birbeck et al., 2010).

The greatest risk of deficits in motor, language and social development is for children younger than 5 years of age (Sierro and Grau, 2019). Three to six months after infection, working memory-specific cognitive deficits can be intensified, with language development being the most consistently affected in pediatric survivors (Idro et al., 2016). Added to this, Carter et al. (2005) demonstrated that after the development of severe malaria, some children present neurocognitive deficiencies that are evident up to nine years later, which become more evident with the children’s age. Children progress and face more complex cognitive and linguistic demands, highlighting that the child’s neurological status at the time of medical discharge was not a good predictor of later neurocognitive impairment (Schiess et al., 2020).

John et al. (2008) demonstrated that cerebral malaria was shown to be associated with long-term cognitive impairment in 1 of 4 surviving children. Newton (1995) demonstrated that of 14 CM unconscious children in Kenya, 6 had cerebral edema and 4 of them showed evidence of ischemic damage associated with critically reduced cerebral perfusion pressure, all 4 of which had severe neurological sequelae. However, even though it is well described in the literature that the development of CM can lead the patient to death and/or induce the development of irreversible sequelae after years of its incidence, the pathogenesis of this condition is still under intense discussion (Newton et al., 1994).

## 3 Pathogenesis of cerebral malaria

The pathology linked to CM exhibits intricate and multifaceted characteristics. Among these elements, the mechanism of cytoadherence of parasitized erythrocytes to the brain microvasculature is emphasized. This process relies on the interaction between antigens found on the surface of parasitized erythrocytes (PfEMP1) and host receptors, including CD36, CD54, ICAM-1 and Endothelial Protein C Receptors (EPCR) (Turner et al., 1994; Storm et al., 2019). In the brain, the adherence of erythrocytes to the endothelium can lead to ischemic processes with the appearance of hemorrhages focus, being the main responsible for the coma state in CM (Berendt et al., 1994). In addition, the production of pro-inflammatory cytokines such as TNF-α and Lymphotoxin α (LT-α) promotes the increase in the expression of ICAM-1, potentiating the sequestration of parasitized erythrocytes, leukocytes and platelets, decreasing the irrigation and oxygenation of various organs of the body, including the brain (Ma et al., 1997; Engwerda et al., 2002; Good et al., 2005; Miller et al., 2002; Hunt and Grau, 2003; Idro et al., 2005).

Studies have confirmed that the increase in the levels of pro-inflammatory cytokines is also a fundamental event for the development and establishment of CM (Brown et al., 1999; Hunt and Grau, 2003). Among these cytokines, TNF-α and Interferon-γ (IFN-γ) secreted by macrophages, CD4+ T and CD8+ T cells stand out. The production of TNF-α in the initial phase of CM seems to be related to the reduction in the parasite load. However, the excessive production of this cytokine in the later phase is associated with the severity of the disease (Omer et al., 2000; Schiess et al., 2020). This divergent role of TNF-α suggests that the regulation and timing of pro-inflammatory cytokine production is of fundamental importance for infection control (Omer et al., 2000).

The release of anti-inflammatory cytokines, mainly interleukin 10 (IL-10) has a protective role by regulating the synthesis of pro-inflammatory cytokines in response to the parasite (Abosalif et al., 2023). Kossodo et al. (1997) demonstrated that the *in vivo* neutralization of IL-10 induces the appearance of the neurological syndrome in animals that were previously resistant to CM, suggesting that this protection mechanism via the release of anti-inflammatory cytokines may be deficient in individuals susceptible to this condition. In this context, the mismatch between the process of releasing pro-inflammatory and anti-inflammatory cytokines seems to be crucial for the development of CM.

In the Central Nervous System (CNS), CM induces damage to the Blood-Brain Barrier (BBB) structure, allowing antigens and inflammatory mediators to enter the brain parenchyma, resulting in microglia activation, neuronal damage, and redistribution of astrocytic cells (Ma et al., 1997; Medana et al., 2001; Adams et al., 2002; Hunt and Grau, 2003). However, the mechanisms involved in BBB alterations, microvascular obstructions, astrocyte degeneration, as well as the homeostasis of neurotransmitter systems, associated with the development of CM still remain poorly understood.

## 4 Blood-brain barrier and cerebral parenchyma alterations in cerebral malaria

The brain tissue is highly sensitive to fluctuations in blood molecules and its homeostasis is meticulously regulated (Menaceur et al., 2021). BBB is a semipermeable membrane that separates peripheral blood from the brain parenchyma, formed by a monolayer of endothelial cells joined by very close junctions and the underlying basal lamina (Nishanth and Schluter, 2019). This structure is considered a part of the neurovascular unit, a concept that emphasizes an interconnection between the different components of the brain for optimal brain functions. BBB integrity is promoted by astrocytes, which are in direct contact with neurons and microglia, being critical to minimizing local inflammation and neuronal damage (Obermeier et al., 2013; Nishanth and Schluter, 2019).

Maintenance of homeostasis is mainly due to brain endothelial cells, which are on the luminal side of the blood-brain barrier and correspond to the actual site of the barrier. They are held together by tight junctions of high electrical resistance, preventing the passage of molecules, and do not contain small openings called cleft pores that allow the diffusion of molecules (Shimizu et al., 2018). Thus, to reach brain parenchyma, essential nutrients need to be actively transported by transport systems to pass through the capillary wall. Brain endothelial cells also have important roles in mediating and regulating the immune response in the central nervous system (Muldoon et al., 2013; Shimizu et al., 2018). The inner part of the BBB is composed by pericytes and glial cells such as astrocytes which work essentially to protect neurons from toxic products released in the bloodstream. In this context, glial cells are responsible for maintaining homeostasis for neuronal functions, contributing and regulating the brain endothelial cell phenotype (Dotiwala et al., 2023). In particular, endothelial cells are in contact with astrocyte foot processes. In several CNS disorders, the dysfunction of the blood-brain barrier is associated with the emergence of substantial neurological conditions such as traumatic brain injury, ischemia and Alzheimer’s disease (Rosenberg, 2012).

During infection with *P. falciparum*, activation of the cerebral endothelium can progress to vascular permeability and loss of BBB integrity, as indicated by hemorrhages in the brains of patients with CM and leakage of dyes or antibodies into the brain parenchyma in experimental cerebral malaria (ECM) (Rénia et al., 2012). Although the extent of pathological events related to BBB function in human CM is variable, BBB dysfunction appears to be associated with brain disease progression (Medana and Turner, 2006). Several studies in patients from Africa and Asia have shown that interruption of BBB in CM leads to serious neurologic complications, including intracerebral hemorrhage, seizures resulting from an electrolyte imbalance, and an increase in intracranial pressure due to generalized edema, resulting in axonal damage, dysfunctions in CNS, activation of glial cells as astrocytes and microglia and neuronal death (Gitau and Newton, 2005; Trivedi and Chakravarty, 2022).

Experimental studies have also illustrated that alterations in the arrangement of astrocytic cells belonging to the blood-retinal barrier contribute to structural impairment and subsequent exposure to vascular contents (MacCormick et al., 2022). Medana et al. (1996) observed a pathological reorganization of retinal astrocytes preceding the development of neurological alterations in ECM. These events precipitate the changes responsible for the retinopathy observed in the progression of CM.

Continuing with vascular alterations in different regions of the CNS, numerous studies indicate that cerebellar atrophy and loss of Purkinje cells, pivotal neurons of the cerebellum, are associated with the characteristic motor manifestations in CM patients (Farahna et al., 2010; Hijazi et al., 2022; Sakaria et al., 2013). Furthermore, cerebellar ataxia after the disease establishment appears to be linked to the cellular loss in this region (Sakaria et al., 2013).

Additionally, recent data have endeavored to elucidate the relationship between changes in glial cells and the pathogenesis of CM. The complication appears to accelerate cellular aging, leading to neuroinflammation resulting from the release of CCL2, CCL5, CXCL9, and CXCL10 from senescent astrocytes. The onset of neuroinflammation can exacerbate the disease by recruiting leukocytes in the brain and reducing neuronal survival during CM (Strangward et al., 2017). The process of senescence in these glial cells may contribute not only to inflammatory changes but also to synaptic impairments, which are responsible for altering the physiology of the CNS.

In response to blood-brain barrier disruption resulting from infections, such as that which underlies CM, a notable alteration in microglial cell activity is often observed. These cells, representing the resident immune system of the CNS, respond to cerebral ischemia and extravasate contents into the parenchyma through the expression of pro-inflammatory cytokines and phenotypic alterations in cellular structure (Medana et al., 2000; Andoh and Gyan, 2021; Shaikh et al., 2024). However, alterations in microglial cell activity can be observed in the early stages of the disease in murine models, which may not be a consequence of tissue changes but rather one of the factors responsible for them. Microglial activation may arise either from direct interaction with parasites or from changes occurring in the microenvironment surrounding the tissue housing parasitic lesions (Mendana et al., 2000).

Indeed, the mechanisms contributing to BBB rupture in cerebral malaria remain elusive. Nishanth and Schlüter (2019) propose several mutually non-exclusive events associated with BBB disorders, as observed in human and murine studies. These include the sequestration of infected red blood cells (iRBCs) to brain endothelial cells (Lau et al., 2015; Ghazanfari et al., 2018), an excessive inflammatory response marked by increased intracerebral pro-inflammatory cytokines (Idro et al., 2010; Lennartz et al., 2017; Trivedi and Chakravarty, 2022) and dysregulation of vascular endothelial cells (Storm et al., 2019). Notably, these events include the involvement of host pathways in tryptophan metabolism and the release of extracellular vesicles, known as microparticles, by both the host and the parasite. Both the sequestration of iRBCS in the vascular endothelium and the aspects of the exacerbated immune response were previously addressed. The clotting process, along with the cytoadherence and the increased host immune response promote brain endothelial cell dysregulation (Storm and Craig, 2014; Plewes et al., 2018). In addition, adjacent factors such as activation and alteration in the distribution of astrocytic cells, activation of microglial cells, as well as pericyte dysfunction and neuronal injury are effects arising from cerebral malaria, which together contribute to BBB degeneration (Dunst et al., 2017). In this context, several other factors must be evaluated, regarding the neurological impairment in CM, making studies in neurochemistry of great importance to bring light to the mechanisms involved in this process.

## 5 Potential mechanisms involved in the development of cerebral malaria

Even among individuals treated with antimalarials and upon recovery, sequelae persist, compromising the functioning of the CNS (Schiess et al., 2020). These sequelae involve a spectrum of challenges, spanning from cognitive impairment and cerebellar-associated motor skill deficits to issues with visual coordination, seizures, and attention deficit hyperactivity disorder. Their impact can extend to up to 25% of pediatric survivors (Idro et al., 2006; Kihara et al., 2009; Schiess et al., 2020), putting them at a heightened risk for deficits in motor, language, and social development. Notably, seizures are a prevalent occurrence in children with CM (Idro et al., 2016; Ouma et al., 2021). Furthermore, as a lasting consequence, sustained seizure disorders may emerge, proving resistant to at least one antiepileptic medication, persisting for months after an episode of CM. Despite numerous studies aiming to elucidate the mechanisms underlying CM development, the pathogenesis of this disease remains poorly understood. Several hypotheses are actively explored to comprehend the intricate elements that contribute to the onset of this complication.

### 5.1 New insights into human and experimental models of cerebral malaria

Unraveling the intricate mechanisms that trigger cerebral malaria is crucial not only for identifying new therapeutic targets but also for comprehending the behavioral disturbances that may arise after an episode of the disease. One of the main limitations of human CM studies is that a detailed analysis of intracerebral pathology can be conducted only after death (White et al., 2010; Ghazanfari et al., 2018). In this context, experimental models in animals have been developed due to the difficulties of evaluating and following up the pathological characteristics in patients with CM.

Between the main experimental models, the murine model is one of the most studied due to the similarities with human CM, mainly in relation to the immune response (Hunt and Grau, 2003; Lacerda-Queiroz et al., 2011). The use of different mice strains, as well as different species of *Plasmodium* shed light on many molecular mechanisms involved in CM (De Souza and Riley, 2002). *P.berghei* ANKA (PbA) strain is most commonly used in experimental models of CM because it activates the sequestration of cells in the microcirculation, triggering a lethal infection at low parasitemia that rapidly progresses to cerebral malaria (Lou et al., 2001; Martins et al., 2009a). Although PbA-induced ECM reproduces some of the features of human CM, the pathology of the disease differs considerably. While human CM is characterized by sequestration of infected red blood cells into the brain microvasculature, with minimal inflammatory changes in the brain, murine ECM shows intracerebral sequestration of iRBCs, with a prominent proinflammatory cytokine response in the brain (Engwerda et al., 2005; Ghazanfari et al., 2018). The infection in susceptible mouse strains leads to the development of CM with main clinical signs of the disease, such as ataxia, respiratory changes and coma (De Souza and Riley, 2002). Recently, Swiss albino mice proved to be a good murine model of CM, showing clinical signs of the disease and histopathological changes in the brain, such as microhemorrhages, cerebral edema and blood flow blockage caused by leukocyte adherence (Martins et al., 2009b; Ataíde et al., 2020). The appearance of these signs varies according to the genetic profile of the host and the strain used, but usually appear between 5-10 days post-infection (De Souza and Riley, 2002). Histopathological studies have demonstrated the occurrence of vessel obstruction, hemorrhage and the migration and adherence of leukocytes to the cerebral vascular endothelium (Gomes et al., 2011). Furthermore, recent human post-mortem reports have revealed intracerebral accumulation of CD8+ T cells, which is also essential for the development of ECM (Rénia et al., 2020; Riggle et al., 2020).

Similar to human cerebral malaria, various authors have extensively characterized the functional alterations induced by cerebral malaria in mice throughout the course of the disease and even after successful therapeutic intervention (Ataíde et al., 2020). Desruisseaux et al. (2008) reported cognitive deficits in mice with cerebral malaria, manifested by a decline in working memory on the 7th day post-infection (d.p.i). This cognitive impairment was concomitant with the presence of hemorrhagic foci and inflammation in the brain parenchyma, as well as the activation of microglia and neuronal degeneration. Dai et al. (2010) described motor and cognitive alterations after treatment with chloroquine in the group infected with PbA. Even after the resolution of hemorrhage and inflammation, there is a necessity for a more detailed examination of the neuronal damage and the impaired synaptic communication in the condition.

In addition, recent studies have demonstrated the emergence of behavioral changes such as cognitive and social deficits induced by milder malaria cases (Guha et al., 2014; de Souza et al., 2018). These findings indicate that even a single episode of mild malaria can induce alterations in the brain cytokine profile, causing specific behavioral dysfunction, accompanied by activation and redistribution of hippocampal microglia and a definitive, but transient, suppression of adult hippocampal neurogenesis (Guha et al., 2014; Schiess et al., 2020; Trivedi and Chakravarty, 2022). Therefore, gaining a comprehensive understanding of the probable mechanisms linked to all the behavioral and neurological changes outlined in this review is crucial. Such insights are for strategically planning therapeutic targets that facilitate the complete restoration of the patient’s quality of life.

As mentioned earlier, liver plays a key role in the biological cycle of *Plasmodium* spp., once the unexpected discovery that the mode of sporozoite entry into the liver profoundly influences the incidence of CM. Sporozoites move from the mosquito bite site to the liver via the bloodstream and exit the liver primarily via Kupffer cells. This is a process mediated by the interaction of the sporozoite surface ligand GAPDH with the CD68 receptor on the Kupffer cell (Loubens et al., 2021; Cha et al., 2015). Studies such as the one by Cha Sung-Jai et al. (2022) corroborate this theory, after demonstrating that CD68 knockout (KO) mice sporozoite liver invasion occurs by breaching the two cells. It was observed that CD68-independent sporozoite traversal induces hepatic vascular injury, which further triggers anti-inflammatory type 2 immune activation for vascular regeneration. Although sporozoite-infected hepatocytes activate IFN-γ mediated inflammation types lining the liver vessels - endothelial and Kupffer, reducing infection by ∼70% compared with wild type (WT) controls.

Plasma assays demonstrated that parasitemia is not the crucial factor for the development of CM condition, but the way in which the sporozoites leave the hepatic circulation, which in turn modulates the host immune response (Ozarslan et al., 2019). No type 2 immunity is activated in CM-positive mice at 2 d.p.i. *In vivo* studies have shed light on the beneficial effects of diets enriched with natural products, notably açaí, renowned for its potent antioxidant and anti-inflammatory properties. Abundant in endemic regions for malaria, such as the Amazon, an açaí-enriched diet has shown significant impacts on the development of vascular, histological and behavioral changes induced by the PbA strain in mice (Oliveira et al., 2022).

Although plasma inflammatory mediators play an important role in the development of CM, others biochemical markers have recently emerged. Lipids and circulating small molecules have a range bioactive functions and a disturbance in their concentrations has been implicated in seizure disorders and other forms of encephalopathy. In the study developed by Gupta et al. (2017) with plasma of human *P. falciparum* infection, it was observed changes in molecules that could impact neurologic function during CM; these include increased levels of kynurenate and decreased indole propionate, glutamate, arginine and glutamine levels. These results highlight the broad changes in blood chemistry during CM and further clinical and mechanistic studies are needed to understand the role of these small molecules on the neurologic impairment and mortality associated to CM.

### 5.2 The role of neurochemical changes in the neurological alterations associated to malaria complications

Seizures and behavioral changes are frequent neurological manifestations of infectious diseases (NMID) caused by viruses, bacteria, fungi and parasites (Kashyap et al., 2023). Metabolic etiologies of seizures are linked to aberrant levels of amino acids, purines, carbohydrates, acylcarnitines and urea cycle defects (Almannai and El-Hattab, 2018; Almannai et al., 2021). Similarly, oxidative stress may play a role in epilepsy development, as compounds possessing antioxidant properties have been shown to mitigate seizure-induced pathology (Shin et al., 2014). Hence, a decrease in molecules that counteract oxidative stress may play a role in the elevated prevalence of seizures and post-CM epilepsy (Shin et al., 2011). Several antioxidants, including indolepriopionate - a neuroprotective free-radical scavenger demonstrated to exert neuroprotective effects in animal model of ischemia - were depleted during CM. Additionally, essential neuromodulators such as glutathione precursors, glutamate, and glycine were also affected (Gupta et al., 2017).

Neurotransmitters and neuromodulators are binding molecules that play a crucial role in cellular communication processes within the Central and Peripheral Nervous System (Rangel-Gomez and Meeter, 2016). The complex network of cellular communication, intricately mediated by signaling molecules and shaped by the interaction between neurons and glial cells within the brain parenchyma, is indispensable for the brain optimal functioning (Rizo, 2018; Zhang and Bramham, 2021). Moreover, this communication network orchestrates essential processes vital for other physiological systems. The complex process of neurotransmission involves several crucial stages, including biosynthesis, packaging, release, receptor interaction, and the subsequent inactivation or uptake of the neurotransmitter from the extracellular environment (Rowley et al., 2012). This nuanced interplay serves as the key to neural communication, and any disruptions within this finely tuned system can be identified as contributors to a wide array of neuropathological conditions (Niyonambaza et al., 2019; Teleanu et al., 2022). Understanding and addressing these disruptions is fundamental in the advancing of our knowledge and to develop effective interventions in the neurobiology of these conditions.

In this context, glutamate emerges as the primary excitatory neurotransmitter in the central nervous system and its interaction with different types of receptors at both pre- and postsynaptic terminals is recognized for its pivotal role in sustaining normal brain function (Yamaguchi et al., 2011). Among glutamate receptors, the notably concentrated expression of the N-methyl-D-aspartate (NMDA) receptor in the hippocampus and cerebral cortex underscores its crucial role in mediating learning, memory, and spatial memory activities within the central nervous system (Kumar et al., 2015). Besides their functional contributions under physiological conditions, both glutamate and NMDA receptors, when overexpressed, have been implicated in the neuropathogenesis of severe degenerative conditions, including the cerebral malaria condition (Singh et al., 2019; Zhang et al., 2019).

#### 5.2.1 Imbalance excitation/inhibition in ECM

Some studies have demonstrated the involvement of glutamate in ECM, as shown by de Miranda et al. (2010), which revealed increased glutamate release in brain cortical regions of C57/BL6 mice infected with the PbA strain, associated with characteristic behavioral changes. As previously described, alterations in retinal tissue are also observed in CM cases. Oliveira et al. (2017) demonstrated an increase in the glutamate uptake from 4th to 7th d.p.i in the retinal tissue. Simultaneously, a reduction in glutathione content, an endogenous neuromodulator with antioxidant properties, was also observed, along with normal levels of TNF-α pro-inflammatory marker normally associated to ECM. This suggests that neurochemical alterations in the retina play a more significant role in CM-related retinopathy than the neuroinflammatory response. de Miranda et al. (2010) additionally demonstrated a significant increase in extracellular glutamate levels on the 7th day post-infection in the cerebral cortex in a murine model of cerebral malaria. In addition, the use of the NMDA receptor antagonist MK801 was shown to promote neuroprotection against changes in long-term memory and depressive-like behavior in PbA-infected mice in a model post-CM (de Miranda et al., 2017). In this work, the neurological changes observed following the treatment, coupled with a normalized inflammatory response and the positive impact of a glutamatergic receptor antagonist, underscore the pivotal role of this crucial neurotransmitter in the manifestation of these neurological disorders. Elevated glutamate levels are known to induce cell death through excitotoxicity, a condition further exacerbated by a diminished endogenous antioxidant response (Atlante et al., 2001; Simões et al., 2018). In pathological conditions characterized by enhanced glutamatergic activity, the involvement of glutamate receptors is pronounced, particularly those permeable to Na^+^, K^+^ and Ca^2+^. Among these receptors, α-amino-3-hydroxy-5-methyl-4-isoxazole-propionate (AMPA)-type glutamate receptors (AMPARs) play a significant role. Additionally, receptors linked to signal transducers, such as metabotropic glutamate receptors (mGluRs), are implicated in the development of neurological alterations observed in the brain during these conditions (Zhang et al., 2015, 2020). Furthermore, members of the mGluRs, belonging to the G-protein-coupled receptor (GPCR) superfamily, are expressed in cells of the neurovascular unit. This expression appears to be crucial for maintaining the integrity of the blood-brain barrier (Mayo et al., 2012; Zhang et al., 2020). Other components of the glutamatergic system have also been implicated in the pathophysiology of cerebral malaria. Evidence from previous studies indicates that treatment with glutamine analog, represented by 6-diazo-5-oxo-L-norleucine, administered during advanced stages of ECM also preserves the BBB integrity and maintains normal neurological parameters, not altering the recruitment of inflammatory cells to the cerebral region (Gordon et al., 2015). Therefore, exploring studies that elucidate the role of the glutamatergic synapse in the context of cerebral malaria holds relevance, given the scarcity of research addressing this pathogenic pathway.

Beyond the pivotal role played by glutamate, other essential amino acids, such as gamma-aminobutyric acid (GABA), are important for maintaining the balance of excitation and inhibition in the central nervous system. The pathogenesis of various neurological disorders unfolds through dysregulations in both GABAergic activity and glutamatergic functions (Ngo and Vo, 2019; Lam et al., 2020). Pioneering work by Sharma et al., 1990 demonstrates that *P. yoelii* infection not only triggers the development of ECM but also induces significant changes in the GABAergic system. These changes are evident through deficits in the activity of glutamic acid decarboxylase (GAD), the enzyme responsible for this neurotransmitter synthesis. Considering that this enzyme plays a direct role in regulating GABA levels, which is critical to control the electrical activity of nervous tissue, these results refine our comprehension between GABAergic dysfunction and the onset of experimental cerebral malaria, highlighting an aspect that has been relatively unexplored. Research indicates enhancements in the cognitive, behavioral and vascular conditions of animals infected with PbA following the administration of neurohormones such as melatonin (Ataíde et al., 2020). Besides its well-established antioxidant and anti-inflammatory properties, melatonin demonstrates a noteworthy ability to interact with GABA receptors, such as GABA_A_. These receptors are responsible for the influx of chloride ions (Cl-) within neurons and glial cells. This interaction has been substantiated by a range of *in vivo* and *in vitro* research, further confirmed through electrophysiological analyses conducted in the hippocampal regions (Cheng et al., 2012; Yu et al., 2023). The studies seem to point to a neuroprotective role for melatonin as a modulator of GABAergic activity. In contrast, deficits in GABAergic activity are commonly observed in other neurological conditions following injuries.

Other factors that may modulate the excitatory activities mediated by glutamate include molecules resulting from the degradation of tryptophan, such as quinolinic acid (QA), kynurenic acid (KA), and picolinic acid (PA). These metabolites can either act as agonists or antagonists of glutamate NMDA receptors, depending on their specific interactions with glycine (Tsuji et al., 2023). Studies have shown an increase in QA levels in the cerebrospinal fluid of young patients in advanced stages of cerebral malaria-induced coma (Medana et al., 2003). KA, acting as an antagonist of glutamatergic activity, appears to exert a neuroprotective effect in injury conditions associated to cerebral malaria. Inhibitors of the enzyme kynurenine 3-hydroxylase, which regulate the QA pathway, have shown promising outcomes in prolonging the survival of mice in experimental cerebral malaria (Clark et al., 2005). Furthermore, this pathway is responsible for the production of other neuromodulators already associated with malaria complications, such as melatonin, as well as neurotransmitters crucial for CNS functions, such as serotonin (Tsuji et al., 2023). Taken together, these findings suggest a significant role for these factors in modulating synaptic signaling during the progression of this disease.

Impairment in other neurotransmission systems, such as the dopaminergic system, specifically linked to an increased expression of the main receptors (D1 and D2 receptors), have been observed in various neurodegenerative conditions, including Parkinson’s disease and in the progression of histological changes associated to ECM (Rangel-Barajas et al., 2015; Kumar and Babu, 2020). The elevated expression of these receptors in the striatal region during ECM appears to be associated with the cognitive and motor changes linked to the condition (Kumar and Babu, 2020). These findings suggest the implication of others neurotransmission systems in this condition.

Further investigations into the dysfunctions of these neurotransmitters systems and their role in the development of neurological disorders in ECM is imperative (**Table 1**). This investigation not only enriches our comprehension of the disease but also harbors the potential to propose innovative medicinal interventions to ameliorate the quality of life for patients affected by this complication.

**Table 1:** Neurotransmitters involvement in HCM and ECM pathogenesis and their potential mechanisms of neuroprotection.

Neurotransmitter System	Mechanisms	References
**Glutamatergic system**	Increased levels of glutamate in CM	de Miranda et al. (2010)
	Alteration in glutamate transport	Oliveira et al. (2017)
	Modulation of glutamate receptor activity: - Administration of NMDA receptor antagonists - Targeting the kynurenine pathway - Other receptor-specific interventions (glutamine)	
		de Miranda et al. (2017)
		Medana et al. (2003); Clark et al. (2005)
		Gordon et al. (2015); Simões et al. (2018)
**GABAergic system**	- Altered synthesis mechanisms	Sharma et al. (1990)
	- Decreased GABA receptor activity	Souza et al. (2018)
**Dopaminergic system**	- Upregulation of dopaminergic receptors	Kumar and Babu (2020)

## 6 Conclusion

**Figure 1:**
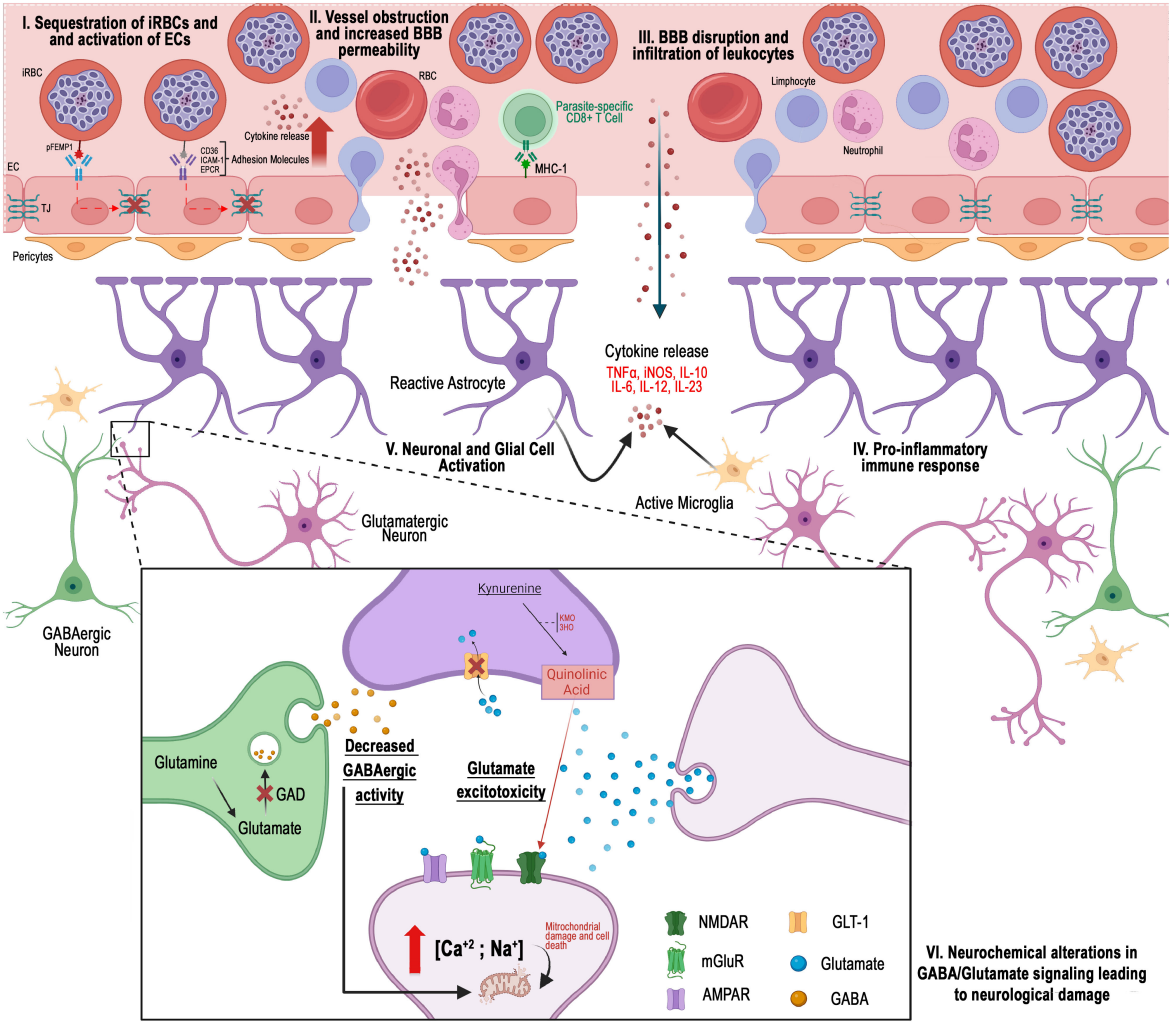
Different perspectives on the pathogenesis of cerebral malaria: I) Hemorrhagic events arise when erythrocytes infected with Plasmodium falciparum become sequestered in the peripheral regions of cerebral blood vessels. II) Cellular obstruction, along with a cytokine-mediated inflammatory response, promotes the increased expression of cellular adhesion molecules. III) The interaction of these cells disrupts the integrity of the blood-brain barrier. IV) Infiltration of vascular content into the cerebral parenchyma occurs. V) Consequently, the function of cells in this region is compromised by the presence of inflammatory and vascular molecules, which may result in neurochemical alterations linked to the development of cerebral malaria. VI) Glutamatergic excitotoxicity, characterized by changes in neurotransmitter levels at the synaptic cleft, reduced GABAergic activity, and the activation of pathways such as the kynurenine pathway, has been implicated in the disease

Cerebral malaria, a deadly complication of *P. falciparum* malaria, has the potential to last cognitive sequelae, even in patients who undergo treatment for the infection. The exposure of the brain parenchyma to vascular and hemorrhagic events, extensively described in the literature, results in tissue disorganization and disruption of communication between neuronal cells, particularly mediated by signaling molecules known as neurotransmitters (**Figure 1**). Recognizing the significance of these molecules in brain physiology, this review highlights studies that offer a new perspective, emphasizing a more crucial role for neurotransmitters in the pathogenesis of cognitive and behavioral changes associated with the disease. Therefore, gaining a better understanding of the neurochemical and physiological effects of the interaction between the parasite and the host becomes crucial in the development of novel therapeutic approaches.
